# Infiltrating the Heart and Kidney: A Rare Pediatric Case of Multisystem Langerhans Cell Histiocytosis from Pakistan

**DOI:** 10.7759/cureus.4315

**Published:** 2019-03-25

**Authors:** Samar Mahmood, Mohammad Raza, Khushboo Nusrat, Shayan Marsia, Awais Abbas

**Affiliations:** 1 Internal Medicine, Dow University of Health Sciences (DUHS), Karachi, PAK; 2 Pediatrics, Dow University of Health Sciences (DUHS), Karachi, PAK

**Keywords:** langerhans cell histiocytosis, pediatrics, oncology, histiocytosis x, multi-system lch, high risk organs, fine needle aspiration cytology, pakistan

## Abstract

Langerhans cell histiocytosis (LCH) is a rare, clonal disease of the monocyte-macrophage system, varying in its clinical presentation from mere self-healing skin and bone lesions to life-threatening multi-system disease. In descending order of frequency, the disease is known to involve the skeleton, skin, lymph nodes and lesser often, the liver, spleen, lungs, hematopoietic and central nervous systems. Here, we present a pediatric case of multi-system LCH in a five-year-old child, unique in its evident cardiac and renal involvement alongside other organ systems and important in how the diagnosis was aided by a fine needle aspiration cytology instead of the costlier histopathological procedures, in a setting with limited resources.

## Introduction

Langerhans cell histiocytosis (LCH) is a rare, clonal disease of the monocyte-macrophage system, characterized by uncontrolled proliferation and accumulation of CD1a^+^/CD207^+^ dendritic cells (DCs) as a result of continuous immune stimulation, resulting in destruction of hard and soft tissues [1]. The proliferation of dendritic cells, in addition to histiocytes, eosinophils, neutrophils and plasma cells is the histological hallmark of this hematological disorder, the neoplastic or inflammatory nature of which remains an unsettled topic of debate [2]. Historically known as “histiocytosis X,” the disease was broadly categorized into three disorders based on the clinical presentations, each of which was staged clinically according to Greenberg et al. clinical staging system before the revised classifications that are used today came to the forefront [3]. The estimated annual incidence of LCH ranges from 0.5 to 5.4 cases per million persons per year, with male predominance and the peak incidence rate being in children aged one to three years old - recorded at three to five cases per million children [4].

The clinical presentation of LCH is highly variable and may range from isolated, self-healing skin and bone lesions to life-threatening multi-system disease, which makes its diagnosis challenging and very essentially aided by the histologic and immunophenotypic examination of tissue lesions [2]. The most common organs to be involved are: skeleton (80%), skin (33%) and lymph nodes (30%) and amongst those less frequently involved are the liver, spleen, lungs, the hematopoietic system and central nervous system (CNS) [2].

Here, we present a pediatric case of multi-system LCH in a five-year-old child, unique in its evident cardiac and renal involvement alongside other organ systems and important in how the diagnosis was made based on a fine needle aspiration cytology - in line with global efforts encouraging its use to achieve a rapid and accurate diagnosis, especially where resources for histopathology are limited as in this case [5].

## Case presentation

A five-year-old boy weighing 11 kg, was brought to a public tertiary care hospital in Karachi, Pakistan by his parents. His chief complaints were described as a diffuse rash over the body, swelling of the head and bulging of both eyes as well as swelling of the gums. These symptoms were progressive and relapsing, with the rash being present since the past 18 months and having extended to a breakout over the head, neck, back, chest and perineum over the last month and the swelling and bulging having progressed over the past year.

According to his mother, the child had been in absolute good health until the age of two years, when he developed a spontaneous fracture of his right clavicle. The family was counseled and told the fracture would heal without any intervention. At two-and-a-half-years of age, the family sought a dental consult for a spontaneously broken tooth which was described to them as a developmental problem of the mandible. Further on, at three years of age, the child developed an infected oral ulcer, a severe swelling of his gums and had two episodes of a yellowish ear discharge for which he underwent drainage. His bodily rash made its first appearance six months after that, at 3.5 years of age, presenting as yellow lesions with pus filled discharge over the head, palms, soles and genital area - albeit, healing afterwards. The swelling of the right side of the head and bulging of both eyes started at the age of four years, with the proptosis gradually progressing to its current state upon presentation and associated with dimness of vision in the right eye. The child had previously been misdiagnosed as a case of Papillon-Lefevre syndrome (rare ectodermal dysplasia characterized by palmoplantar keratoderma associated with early onset periodontitis) two years ago and prescribed fluconazole, but to no avail. He was the third offspring of a consanguineous marriage, fully vaccinated and developmentally appropriate.

On general examination, the child was conscious and well oriented, although he had had a single episode of a minute long, tonic seizure, 15 days back. He appeared underweight and shorter than the desired height for his age. He was in respiratory distress, with a respiratory rate of 50 per minute, harsh vesicular breathing and prominent bilateral crepts. His apex beat was shifted and there was a notable gallop rhythm in the heart sounds. Gastrointestinal examination showed a distended abdomen with prominent veins and a palpable liver (5 cm below the right costal margin) and spleen. He was moderately anemic, had grade 3 clubbing and moderate, bilateral, pitting, pedal edema. There was no cyanosis or jaundice. The cervical submandibular lymph nodes were palpable, as was a single node in the left axillary region - each being 1 cm in size.

**Figure 1:**
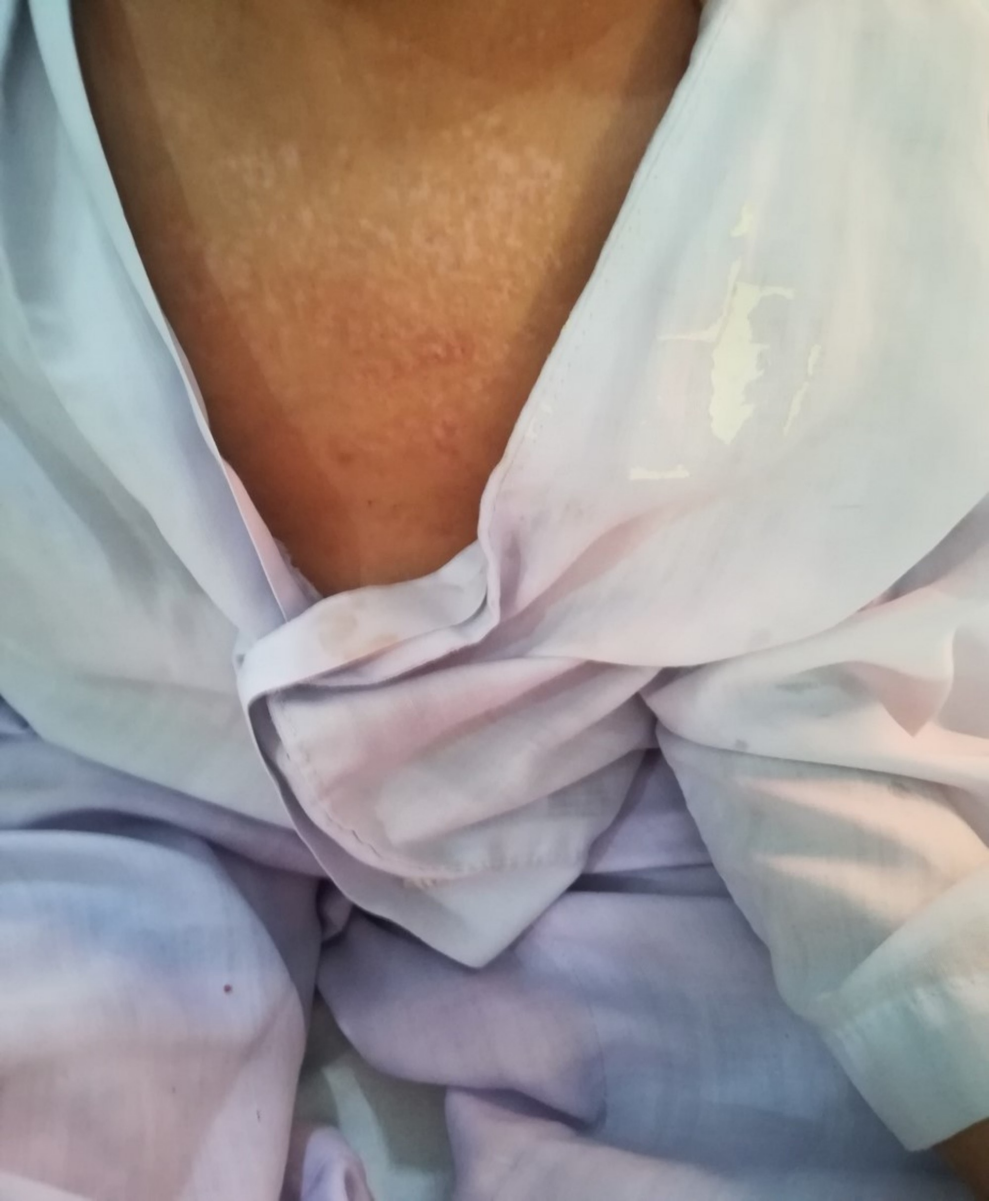
Lacy depigmented rash after healing.

Upon local examination: the head showed frontal and parietal swellings - each measuring about 5 cm by 4 cm and nontender on palpation. The ophthalmic examination showed bilateral proptosis, more on the right and with a hazy cornea, but with both pupils reactive to light. The oral cavity examination showed a gingival swelling. The skin rash appeared to be scaly, papular seborrheic dermatitis - mainly on the scalp, neck, back, palms, soles and genital area, covered with pustules and having progressed to a lacy depigmented rash after healing, in some places (Figure 1).

Laboratory investigations revealed a hemoglobin of 6.6 g/dL, white blood cells - 29.7/mm^3^ (56% neutrophils, 39% lymphocytes), platelets - 113 x 109/L and a serum ferritin was 362 ng/ml which was significantly higher than the range for his age. The ultrasound of the abdomen exhibited an enlarged liver, bilaterally enlarged and moderately hydronephrotic kidneys and minimal pelvic ascites, without any enlarged lymph nodes. The echocardiography showed an enlarged left ventricle with generalized dysfunction and an ejection fraction of 35%. His computed tomography (CT) scan revealed an evident ground glass haze in the lungs, with thickening of skull bones, especially at the base of skull and bilateral sphenoid bones that raised the possibility of fibrous dysplasia. However, the septal thickening with ground glass haze noted in both lung fields, cardiomegaly, hepatomegaly (16.5 cm) and bilateral hydronephrosis - all suggested a more widespread, multi-system involvement, raising the possibility of LCH.

**Figure 2:**
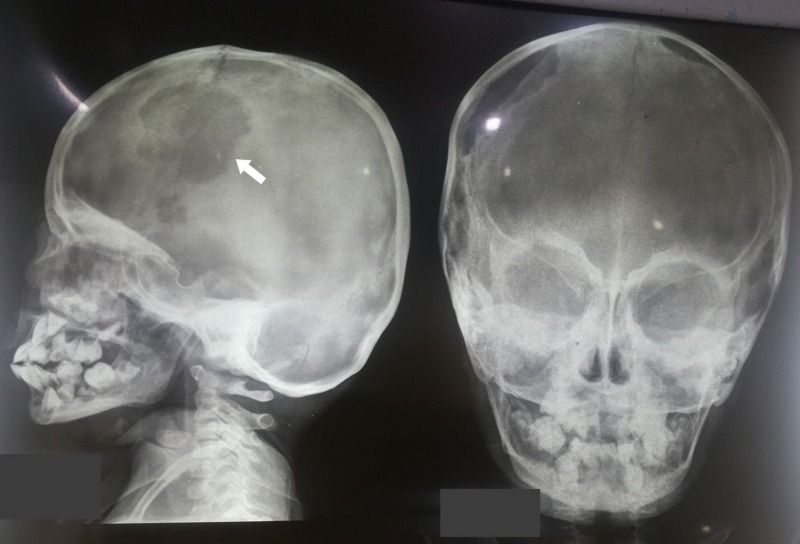
X-ray showing lytic lesion in the skull.

Further, the magnetic resonance imaging (MRI) of the brain and orbits showed evidence of a focal lesion, causing an erosive destruction of the calvarium within the frontoparietal region on the right side, 3 cm by 6.5 cm and another lesion causing erosive changes within the superior parietal calvarial region (the lytic lesions can be seen in the X-ray attached as Figure 2). Both these lesions portrayed intracranial invasion with diffuse infiltration of meninges and compression over the cerebral cortex. Additionally, lobulated meningeal enhancement could be seen along the tentorial region bilaterally. There was diffuse and bilateral enlargement of the extraocular muscles, more marked on the right side, with involvement of the lacrimal glands. Due to limited financial resources, a bone marrow biopsy could not be ordered but a fine needle aspiration cytology (FNAC) was carried out and reported numerous multinucleated cells, foamy phagocytic histiocytes and few polymorphonuclear cells. Based on these findings, a diagnosis of malignant histiocytosis, specifically LCH, was made.

The child was started on etoposide (150 mg/m^2^) and prednisone (40 mg/m^2^/day), alongside supportive oxygen inhalation, broad spectrum antibiotics, antifungals, inotrope support, treatment for heart failure with IV furosemide and oral captopril, parenteral nutrition, a xanthine oxidase inhibitor and calcium carbonate in tablet form. He responded well to this treatment - his cardiac and renal status improved and blood pressure normalized. He is currently on the continuation phase of his treatment and regularly follows up at the oncology ward.

## Discussion

Langerhans cells, the histiocytes reportedly present in lesions of LCH, are dendritic mononuclear cells normally found in the epidermis, mucosa, lymph nodes and bone marrow. Their normal function is to process and present antigens to T-lymphocytes; however, in LCH, there appears to be a monoclonal proliferation of these cells, leading to destruction of hard and soft tissues and pointing towards the neoplastic nature of the condition [6]. Historically categorized as Eosinophilic granuloma, Hand-Schuller-Christian disease, and Letterer-Siwe disease under the term ‘histiocytosis X,’ LCH is now divided into three types by a recognized classification method, including ‘acute disseminated,’ ‘chronic multifocal’ and ‘chronic focal LCH.’ However, it is more commonly divided into two broad types, according to the lesion scope. These include ‘localized LCH’ - which is defined by a simplex rash, without affecting organs and simplex or multiple bone damage, with or without diabetes insipidus (DI), adjacent lymph nodes involvement or rash - and ‘disseminated LCH’ - where the internal organs are involved, with or without DI, adjacent lymph nodes involvement, rash and dysfunction of lung, liver or hematopoietic system [7]. Importantly, although LCH is not considered a hereditary or familial disease, surveys within twin-patients and in same generation, non-twin familial LCH have pointed to a subset of the disease that mimics a heritable pattern of inheritance. Of relevance, all ancestors of families with multiple patients of LCH in a single generation came from very circumscribed rural areas, suggesting possible consanguinity, as was seen in our case as well [8]. This finding lends support for an autosomal recessive pattern of inheritance in a subgroup of patients with LCH, an aspect of the disease that requires further research.

Regarding presentation, LCH has a wide clinical spectrum and its clinical course is essentially determined by the age of the patient and pathological infiltration of Langerhans cells in the various systems. While adults and older children usually present with single system disease commonly affecting the bone, younger babies demonstrate multisystem involvement - where the initial general clinical picture includes skin rashes, otitis media, fever, organomegaly, anemia, pain and pathological fracture of involved bone - as typically seen in our patient, since he was two years old - and sometimes, diabetes insipidus as well. Although sparse, the existent literature about cases in adults repetitively mentions lesions of the oral cavity as the first and occasionally, the only manifestation [9]. This oral involvement is mostly seen as gingival hypertrophy, oral ulceration, mobility of teeth with alveolar expansion, jaw pain, facial swelling, and mental nerve anesthesia [10]. Furthermore, the skeletal involvement due to osteolysis by the infiltration of Langerhans cells may occur in any part of or whole bone, however, in the head and neck region, it is commonly seen in the mandible - as seen in our case [9]. Cutaneous lesions vary from crusted nodules and papules, blisters, vascular tumor-like lesions, scaling orange to red macules (frequently in seborrheic regions) to purpuric macules; later onset and a more protracted course of skin lesions are reportedly more frequent findings in multisystem disease [11]. Interestingly, despite these numerous characteristic symptoms, LCH may very well be asymptomatic or present only with non-specific symptoms such as fever, poor appetite, weight loss, fatigue, irritability and changes in behavior [12]. This extreme variability coupled with the rarity of the condition make its diagnosis a challenging task.

Disease involvement of organs in LCH is considered as a marker of higher risk of either dying from disease (risk organs) or developing neuro-degenerative complications, more commonly named as CNS risk lesions. The hematologic system, spleen, liver and debatably, the lung, are considered risk organs whereas skull bone lesions, CNS involvement, orbital and ear involvement mark a greater risk of neuro-degenerative complications [13]. Cardiac and renal involvement, as seen in our case, are both extremely rare in LCH - even more so in children. Gholami reported a case of pericardial effusion in a seven-month-old girl, in 2016, stating that just two other LCH cases in the existing literature mentioned the same [14]. Simmons and Rosamond reported a rare diagnosis of fibroxanthomatous infiltrative myocardial disease due to LCH in a 44-year-old in 2009 [15], which highlights how LCH must be kept on the list of differentials for infiltrative myocardial diseases in adults. However, our findings of an enlarged left ventricle with generalized dysfunction, an ejection fraction of 35%, a shifted apex beat and prominent gallop rhythm in a five-year-old LCH patient, differed from previous reports and warrant further study. In contrast, renal involvement in LCH is relatively more reported, having previously presented as interstitial nephritis, membranous glomerulonephritis, acute renal failure due to the renal parenchymal infiltration by histiocytes and renal amyloidosis in adults and minimal change nephropathy in children [16].

Furthermore, the diagnosis of LCH is clinicopathologic and should only be made in the appropriate clinical setting to prevent misdiagnosis in the presence of normal reactive Langerhans cells, particularly in regional lymph nodes. In addition to clinical and radiological features, LCH diagnosis must always be confirmed only after histological and immunophenotypic examination of lesional tissue - taken from the most easily accessible, yet representative lesion [13]. A definitive unequivocal diagnosis requires demonstration through electron microscopy of intracytoplasmic ‘Birbeck bodies’ or immunostaining of the cells by anti-CD1a and anti-S100 antibodies, with negativity for anti-CD68 along with positive CD1a. Langerin (CD-207) is another useful highly specific Langerhans cell marker [17]. Additionally, fine needle aspiration (FNA) can be used to establish the extent of disease or recurrence of LCH. Since the cytologic features of LCH are highly characteristic to suggest a diagnosis in an appropriate clinical setting with classical radiological findings, this increases the role of FNA in the diagnosis of LCH in children with usual clinical presentation. This can obviate the need of biopsy and electron microscopy with immunochemistry being performed on cell blocks prepared from the residual aspirates as a useful adjunct [5]. This is proving to be of specifically useful in third world countries like Pakistan, where the patients have limited financial resources, as in our case.

Finally, the treatment strategies for LCH differ based on the age of the patient, type of disease and are focused on the organ(s) predominantly involved. The major clinical challenges of multisystem-LCH (MS-LCH) are mortality in young children with involvement of risk organs, and bouts of reactivation resulting in morbidity and permanent consequences which can occur in all age groups. Several international protocols for MS-LCH treatment have been designed, mainly concluding that: standard treatment is based on steroids and vinblastine (VBL), clinical response after the first six weeks of treatment is a good marker of further disease evolution and prolonged treatment for at least one year reduces the risk of disease reactivations. Refractory disease in patients with hematological involvement or liver dysfunction requires referral to specialized centers, rare cases with a potential risk of developing a malignant tumor are advised radiotherapy and the neurodegenerative complications are addressed by a multi-disciplinary team, treated via: retinoic acid, combination treatment with vincristine and Ara-C, intravenous immunoglobulin, and cladribine. Regarding follow-up in children, it is usually advised till (i) at least five years after the end of therapy; or (ii) five years after the last disease reactivation, in those who did not receive systemic therapy; or (iii) until final growth and pubertal development have occurred [13]. The prognosis of LCH varies depending on its form, with that for patients with single-system single site LCH being the relative best. At present, the factors for poor prognosis include age under two years old, multi-system disease, high-risk organ involvement and dysfunction or a poor response to initial therapy. Importantly, hepatic involvement at the time of the diagnosis is particularly associated with a poor prognosis [12].

## Conclusions

Thus, we see a rare case of Langerhans cell histiocytosis in a five-year-old child with multi-system involvement of various high and low-risk organs and the associated clinical presentation, laboratory findings, classification criteria, considered differentials, treatment and prognosis. This article highlights the need for future work to be done regarding the autosomal recessive pattern of inheritance in a subset of the disease, regarding the cardiac and renal involvement reported and the efficacy of FNAC as an independent diagnostic investigation for the condition - all of which will enable better understanding and improved, earlier diagnoses.
